# Response to letter to the editor PD‐17‐0390, a comment on “Comparing methods for fetal fraction determination and quality control of NIPT samples”

**DOI:** 10.1002/pd.5170

**Published:** 2017-12-21

**Authors:** Daphne M. van Beek, Roy Straver, Marjan M. Weiss, Elles M.J. Boon, Karin Huijsdens‐van Amsterdam, Cees B.M. Oudejans, Marcel J.T. Reinders, Erik A. Sistermans

**Affiliations:** ^1^ Department of Clinical Genetics VU University Medical Center Amsterdam Amsterdam The Netherlands; ^2^ Department of Clinical Genetics Leiden University Medical Center Leiden The Netherlands; ^3^ Department of Clinical Genetics Academic Medical Center Amsterdam The Netherlands; ^4^ Department of Clinical Chemistry VU University Medical Center Amsterdam Amsterdam The Netherlands; ^5^ Delft Bioinformatics Lab Delft University of Technology Delft The Netherlands

In a recent edition of *Prenatal Diagnosis*, we published a comparison between bioinformatic methods that estimate the fetal cell‐free DNA fraction in maternal plasma, which is essential knowledge when performing NIPT tests.^1^ Grendar et al confirmed the need for this paper, but commented on our findings by stating that we left data unexplored, mainly pointing towards observable biases between the different approaches (ie, deviation from the 45^o^ line through the origin in pairwise comparisons).^2^ Clearly, these systematic differences can be observed (an updated figure showing this effect more clearly, as well as a table containing the underlying data are included as new supplementary data). However, since there is no gold standard available (ie, the true fetal fraction is unknown), nor is it (easily) faithfully extractable, it is not possible to confirm which bias is to be preferred. Therefore, we decided not to zoom in on this level of comparison, but to restrict to conclusions of a more general nature.

Mainly, we compared three methods: Y chromosome–based methods (DEFRAG, BAYINDR), a different method based on all chromosomes (SeqFF), and one based on fragment lengths of the cell‐free DNA (SANEFALCON). As Y‐based methods for pregnancies with male fetuses are likely closest to the truth (due to the absence of the Y in the mother), looking at correlations with the no‐Y‐based methods makes sense (this is also not argued by Grendar et al). This comparison shows that the no‐Y‐based methods are more variable, and consequently have a lower correlation to the Y‐based methods. Yet, in contrast to Y‐based methods, they can still be used for pregnancies carrying female fetuses, as their correlation is still good (>0.9 for SeqFF). Within the Y‐based methods, we also compared whether focusing on uniquely mapping male regions on Y would increase reliability (differences between the a/b methods for both DEFRAG and BAYINDIR). BAYINDIR is especially sensitive for this difference, with BAYINDIRa (not focusing on male regions) having the largest variations in addition to nonzero fetal fractions for female pregnancies. We therefore concluded that focusing on uniquely mappable Y regions is preferable. The correlation between DEFRAGb and BAYINDIRb is high (0.984), resulting in our main conclusion to use any Y‐based method for male‐pregnancies. Zooming in on the differences between both methods, we see a minor systematic difference, with DEFRAGb resulting in slightly higher estimates of the fetal fraction than BAYINDIRb (Figure 1 in our manuscript). As stated before, in the absence of a gold standard, there is no way to tell which one is better, which prompted us to the more general conclusion on using any Y‐based method. Grendar et al, claim that these systematic differences (on the order of 2.5%) make a huge difference when used in the NIPT procedure. Granted, if we would have implied that the estimates are unbiased estimates of the real fractions, this difference is important. But, we did not claim this. In fact, we argue that the remark by Grendar et al is deceptive. First, the difference is artificial (when not knowing the true values). Second, and more importantly, in practice one can easily adapt decision thresholds to these differences. For NIPT this would result in shifting the decision threshold with 2.5% between the two methods when deciding on having enough fetal DNA to reliably interpret NIPT results. Taken together; the claims we made are supported by the correlations; systematic biases are present as Grendar et al rightly pointed out, but they cannot be interpreted and can easily be accounted for in clinical practice; the accuracy of the estimates of the fetal fraction is not known due to lack of a gold standard, but for clinical practice this is not essential.

**Figure 1:**
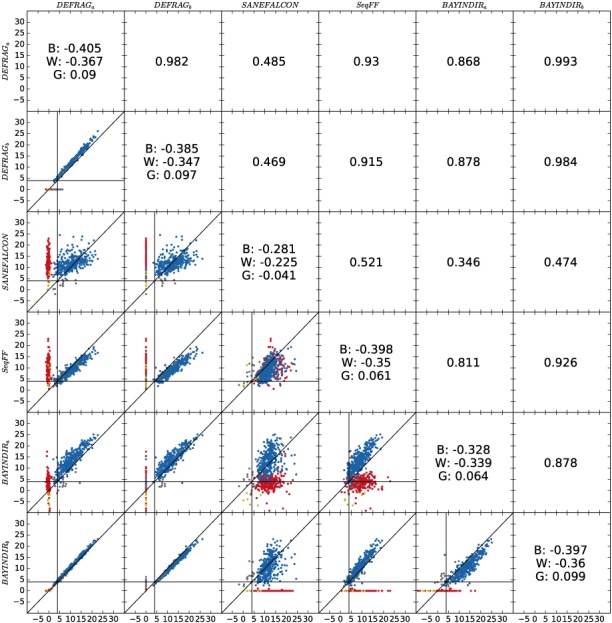
The lower left part of the matrix shows our comparison of the 6 different methods to predict fetal fraction from single‐read NGS data for 654 maternal blood plasma samples. Blue dots represent the male pregnancies; red dots, the female pregnancies. Gray and green dots represent male and female pregnancies of a failed run, which contained degraded fetal DNA. On the diagonal of the matrix, the correlations to BMI (B), weight (W), and the gestational age (G) are shown, respectively. A diagonal line has been included in all lower left panels to indicate systematic differences. The upper right part of the matrix shows the correlation between the 2 methods [Colour figure can be viewed at wileyonlinelibrary.com]

## Supporting information

Data S1. Supporting Information
